# Selectivity control towards CO versus H_2_ for photo-driven CO_2_ reduction with a novel Co(II) catalyst

**DOI:** 10.3762/bjoc.19.129

**Published:** 2023-11-17

**Authors:** Lisa-Lou Gracia, Philip Henkel, Olaf Fuhr, Claudia Bizzarri

**Affiliations:** 1 Institute of Organic Chemistry (IOC), Karlsruhe Institute of Technology (KIT), Kaiserstrasse 12, 76131 Karlsruhe, Germany; 2 Institute of Nanotechnology (INT), Karlsruhe Institute of Technology (KIT), Kaiserstrasse 12, 76131 Karlsruhe, Germany; 3 Karlsruhe Nano Micro Facility (KNMFi), Karlsruhe Institute of Technology (KIT), Kaiserstrasse 12, 76131 Karlsruhe, Germany

**Keywords:** carbon monoxide selectivity, cobalt(II) complex, copper(I) complex, earth-abundant, hexafluoropropanol, photocatalytic CO_2_ reduction

## Abstract

Developing efficient catalysts for reducing carbon dioxide, a highly stable combustion waste product, is a relevant task to lower the atmospheric concentration of this greenhouse gas by upcycling. Selectivity towards CO_2_-reduction products is highly desirable, although it can be challenging to achieve since the metal-hydrides formation is sometimes favored and leads to H_2_ evolution. In this work, we designed a cobalt-based catalyst, and we present herein its physicochemical properties. Moreover, we tailored a fully earth-abundant photocatalytic system to achieve specifically CO_2_ reduction, optimizing efficiency and selectivity. By changing the conditions, we enhanced the turnover number (TON) of CO production from only 0.5 to more than 60 and the selectivity from 6% to 97% after four hours of irradiation at 420 nm. Further efficiency enhancement was achieved by adding 1,1,1,3,3,3-hexafluoropropan-2-ol, producing CO with a TON up to 230, although at the expense of selectivity (54%).

## Introduction

Solar energy conversion into chemical energy addresses the issues of energy shortage with the exploitation of renewable sources [1]. Photoinduced CO_2_ reduction is included in the vast research field of artificial photosynthesis. Taking Nature as a model, the absorption of photons can drive electron-transfer processes, leading to the production of highly energetic molecules. By aiming at the conversion of CO_2_, a greenhouse gas implicated in climate change, the closure of the carbon cycle can be achieved [2]. For this purpose, three main components are needed: a photosensitizer (PS), which acts like a light-antennae harvesting system in natural photosynthesis, a catalyst (Cat.), reacting directly with CO_2_ after being reduced, and a sacrificial electron donor (SeD). When the involved (photo)catalysts are homogeneous transition-metal-based complexes, the outcomes are generally two-electron reduction products, such as carbon monoxide (CO), formic acid (HCO_2_H), or formate (HCO_2_^−^). To mitigate the strong energetic requirements of the reaction shown in Equation 1, the reduction of CO_2_ occurs in the presence of protons, so that the energy barriers of the reactions shown in Equation 2 and Equation 3 are lowered.


[1]

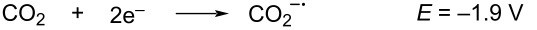




[2]

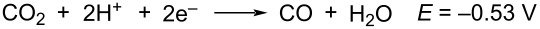




[3]

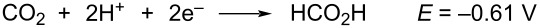




[4]

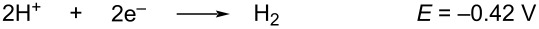



In fact, the formation of the radical anion CO_2_^−^**^·^** takes place at −1.9 V versus normal hydrogen electrode (NHE), while the proton-assisted reductions of CO_2_ to CO and formic acid happen at −0.53 V and −0.61 V (versus NHE), respectively [3]. However, the molecular hydrogen evolution might compete, as it occurs at a more favorable reduction potential, lowering the selectivity of the catalytic system. While the addition of a proton source is beneficial to lower the overpotential, a metal-hydride (M–H) intermediate could be favored concerning the formation of the CO_2_ adduct with the reduced metal center. Thus, besides the development of novel efficient catalysts, different strategies have been pursued to switch the catalyst selectivity towards carbon products [4–5]. Generally, scientists can interplay by developing the major components of a photocatalytic system for CO_2_ reduction, such as the photosensitizer (PS), the catalyst, and the sacrificial electron donor (SeD). Nevertheless, the solvent and eventual additives play an important role too [6], as they can influence the (photo)redox properties of the major components, fostering or dropping the efficiency. Thus, a rational design of novel molecular catalysts should consider an additional development of the whole system [7]. Moreover, it would be beneficial for future applications, if major efforts are focused on earth-abundant materials [8–11]. Among the most employed earth-abundant metal-based PS, Cu(I) complexes have the first place, not only in artificial photosynthesis, but also in a large variety of photo(redox)catalyses [12–17]. On the other hand, several complexes based on 3d transition metals, like manganese [18], iron [19–21], cobalt [22–23], and nickel [24–25], have been designed as CO_2_ reduction catalysts. This (supra)molecular approach is appealing for gaining a structure–property understanding with the goal of tunable and efficient activity.

Among the 3d transition metals, cobalt is relatively abundant (26.6 ppm) in the Earth crust [26]. Although it should not be considered a cost-effective option at present, as several social and environmental concerns are associated with its extraction, the high stability of the Co(II) ion and the versatility of the ligands used for coordination offer some advantages for tailoring new catalysts to specific reactions and optimize selectivity [22,27]. Cobalt catalysts successfully employed in CO_2_ reduction are mainly based on macrocyclic ligands, such as tetraazacyclodecene and its derivatives [3,28–29], porphyrins [30–34], or phthalocyanines [35]. The use of bimetallic complexes has resulted in a favorable mechanism, increasing yields tremendously [36–38].

**Figure 1:**
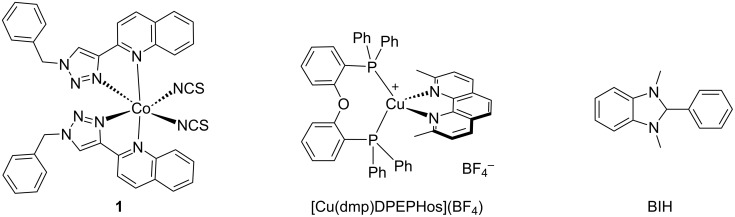
Chemical structures of the molecular components used in this work: Co(II) complex **1** as the novel catalyst, the heteroleptic Cu(I) complex as photosensitizer, and the benzimidazolidine derivative BIH as the sacrificial electron donor.

Targeting efficient completely earth-abundant metal-based systems, we have designed a novel Co(II) catalyst for the reduction of CO_2_ (complex **1** in Figure 1). The design aimed at a stable complex obtainable via a straightforward synthesis, with improved solubility, concerning our previous Co(II) complexes [21]. Thus, the new Co(II) complex bears two 1-benzyl-4-(quinolin-2-yl)-1*H*-1,2,3-triazole (BzQuTr) units, that were obtained through a copper-catalyzed alkyne–azide cycloaddition (CuAAC) [39–40], and two thiocyanate ligands. As observed for other cobalt complexes [21], the photoinduced CO_2_ reduction gave preferentially molecular hydrogen, when performed in acetonitrile. Moreover, we targeted a photocatalytic system that is fully earth-abundant. For this reason, we selected the known complex [Cu(dmp)DPEPhos](BF_4_), well-investigated and used in several photocatalytic reactions [20–21,41], acting as a cost-effective benchmark photosensitizer. Herein, we present a study for the selectivity control of the novel Co(II) catalyst **1**, aiming at maximizing the catalytic efficiency, and maintaining high selectivity for carbon products.

## Results and Discussion

### Synthesis and characterization of the new Co(II)-based catalyst

**Figure 2:**
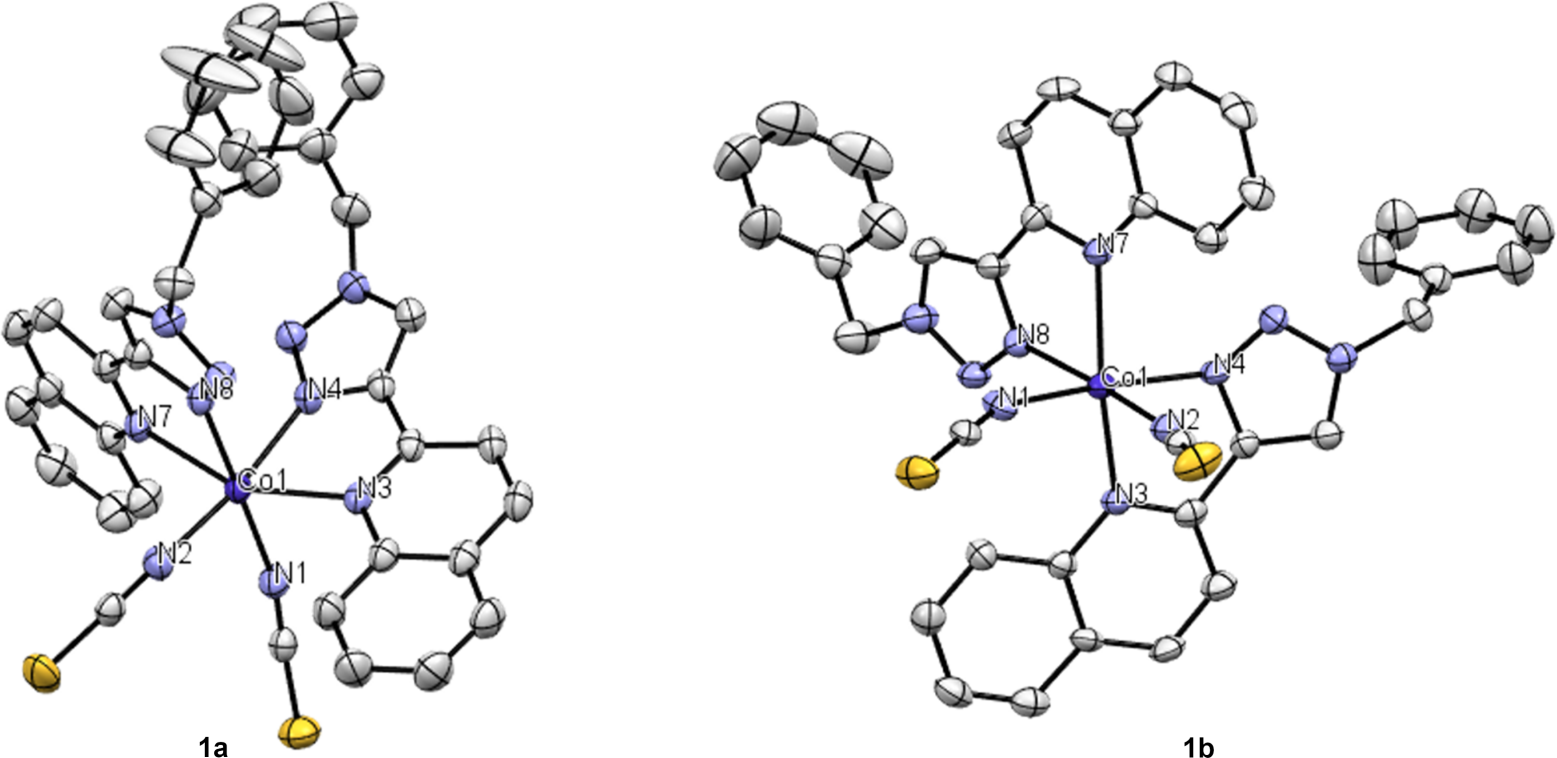
ORTEP drawing of crystal polymorph **1a** (left) and **1b** (right), shown at the 50% probability level. Hydrogen atoms and co-crystallized solvent molecules are omitted for clarity.

The novel cobalt(II) complex **1** was synthesized in dry methanol (MeOH) by mixing in a 2:1 ratio, the chelating diimine ligand, 1-benzyl-4-(quinolin-2-yl)-1*H*-1,2,3-triazole (BzQuTr) [42] and the cobalt precursor Co(NCS)_2_(py)_4_ [43], where py is pyridine. The reaction was performed under an argon atmosphere at room temperature. The resulting complex **1** was obtained after evaporation of the solvent, as a lilac precipitate in good yield (60%). The structure was investigated by high-resolution mass spectrometry (ESI), where it was shown the fragment corresponding to complex **1** that lost one isothiocyanate, [M − NCS]^+^, as the primary signal. Elemental analysis matched the calculated values, incorporating an additional MeOH molecule. Recrystallization was afforded by re-dissolving the powder in acetonitrile and layering on top of diethyl ether (Et_2_O). Slow diffusion of the antisolvent Et_2_O allowed the growth of magenta-colored crystals. Interestingly, two different sets of data could be solved, which is an indication that compound **1** has two polymorphs, **1a** and **1b** (Figure 2). Efforts to selectively achieve one polymorph, through differentiated crystallization processes, were unsuccessful. When analyzing the molecular structure in both crystals, the cobalt core is hexacoordinated, as expected. The two isothiocyanate ions are oriented *cis* to each other and *trans* to the coordinating nitrogen of the 1,2,3-triazole units. The nitrogen atoms of the two quinoline moieties are therefore *trans* to each other. This conformation might be induced by the cobalt precursor Co(NCS)_2_(py)_4_, which has already the NCS monodentate ligands *cis* to each other, as it was not the case for other Co(NCS)_2_(NN) complexes, where NN is a chelating diimine compound such as pyridyl-tetrazole [44], or a pyridine-oxazole [45]. The bond lengths are very similar when comparing the polymorphs **1a** and **1b**. Nevertheless, the bond angles vary significantly (see Table S2 in Supporting Information File 1). Polymorph **1a** crystallizes with two molecules of acetonitrile in a triclinic system, while **1b** contains one CH_3_CN molecule and has an orthorhombic crystal system. The lattice packing of the two polymorphs with solvent molecules is shown in Supporting Information File 1 (Figures S1 and S2). We were not able to detect, if the two polymorphs show different catalytic activity, as in the following investigations the amorphic powder was used.

### Spectroscopic and electrochemical characterization

**Figure 3:**
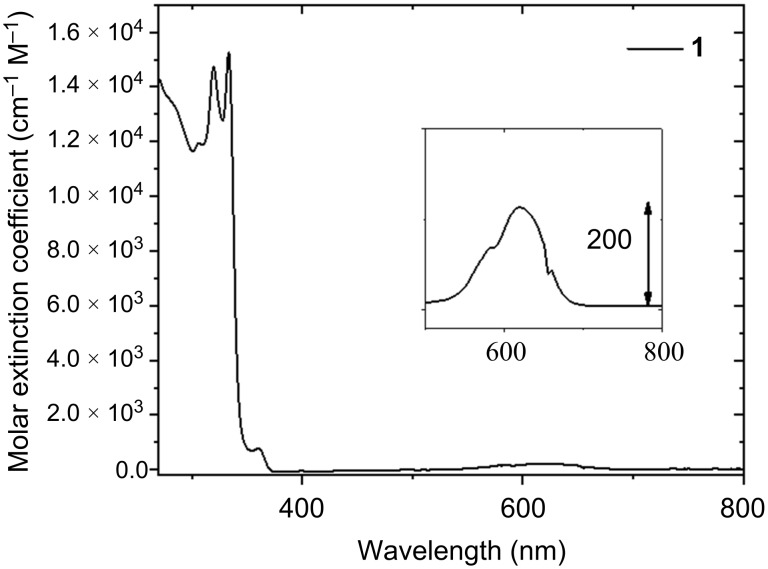
UV–vis absorbance of complex **1** in DMA. Inset: zoom-in of the 500–800 nm range to visualize the low-intensity bands associated with metal-centered d–d transitions.

The Co(II) complex **1** was characterized by UV–vis absorption spectroscopy in *N,N*-dimethylacetamide (DMA), as it was the chosen solvent for photocatalysis. The absorption profile evokes the structured band of the free ligand BzQuTr [42], with two intense π–π* ligand-centered transitions at circa 319 nm and 330 nm (Figure 3). The pink solid dissolves as an intense blue DMA solution. Nevertheless, the d–d transitions associated with this absorption centered at 615 nm possess a low molar extinction coefficient (ε ≈ 220 cm^−1^ M^−1^, inset in Figure 3). Infrared (IR) spectroscopy was performed via attenuated total reflectance (ATR) and showed the characteristic stretching vibration of the NCS groups at 2069 cm^−1^ (Figure S3 in Supporting Information File 1).

**Figure 4:**
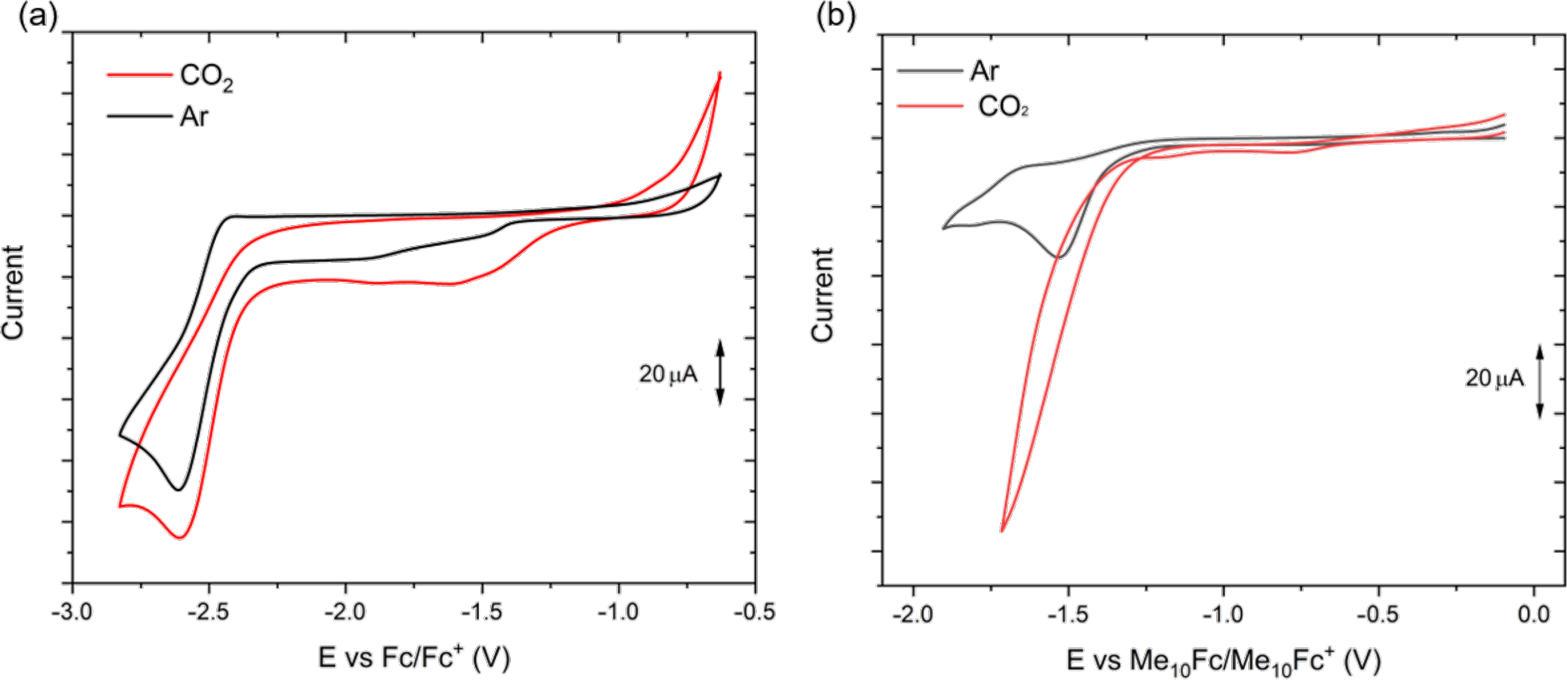
Cyclic voltammetry of complex **1** in 0.1 M TBAPF_6_ solution of (a) DMA and (b) DMA/TEOA 5:1 (v/v). Black curves are registered under Ar and red curves are recorded under CO_2_ atmosphere. A glassy-carbon disk was used as the working electrode and the internal references used are (a) ferrocene and (b) decamethylferrocene. Scan rate was 100 mV s^−1^.

The redox properties of **1** were investigated using cyclic voltammetry in a DMA solution with 0.1 M tetrabutylammonium hexafluorophosphate (TBAPF_6_) as the supporting electrolyte (Table 1). The concentration of the analyte was 5 mM. Only the cathodic scan resulted in a rich profile of redox processes (Figure 4a, black lines). In particular, two irreversible reductions occur at the cathodic potentials −1.53 V and −1.9 V versus ferrocene. These electrochemical processes may correspond to the first and second reduction of the metal core Co(II)/Co(I) and Co(I)/Co(0), respectively. A more intense current arises with the third redox process occurring at −2.52 V, which could be assigned to the reduction localized on the ligand (compare with the cyclic voltammogram in Supporting Information File 1, Figure S8).

**Table 1:** Optical and electrochemical properties of complex **1** in DMA.

λ_abs_, nm	ε, M^−1^ cm^−1^	*E*_red_,^a^ V	*E*_red_,^b^ V

319	14720	−1.53	−1.56
333	15257	−1.90	−2.02
615	220	−2.52	

^a^In 0.1 M TBAPF_6_ solution of DMA, versus Fc/Fc^+^ potential; ^b^in DMA/TEA 7:1, 0.1 M TBAPF_6_ versus Me_10_Fc/Me_10_Fc^+^.

We investigated the electrochemical properties also under a CO_2_ atmosphere (Figure 4, red curves). In DMA, the electrochemical behavior changed only moderately, suggesting that the reduced complex **1** does not react promptly with carbon dioxide under these conditions. On the other hand, when a 5:1 mixture of DMA/triethanolamine (TEOA) was used as the solvent, a significant catalytic current was observed at the onset potential of −1.4 V. Although a direct comparison between Co(II) and Fe(II) ions cannot be made, it is reasonable to suggest that after the first reduction a ^−^NCS ligand detaches and an adduct with CO_2_ is formed, as it was calculated for a similar thiocyanate-based Fe(II) complex [46]. These results suggested that cobalt complex **1** can be used in CO_2_ reduction reactions (CO_2_RR).

### Photo-driven CO_2_ reduction

Next, we explored catalyst **1** in the CO_2_ reduction via photoirradiation. A well-known Cu(I) complex was selected as a photosensitizer since we were interested in the development of earth-abundant systems. In particular, we chose the heteroleptic complex [Cu(dmp)DPEPhos](BF_4_), where dmp is 2,9-dimethyl-1,10-phenanthroline and DPEPhos is bis[(2-diphenylphosphino)phenyl] ether, which had been already successfully employed in other artificial photosynthesis [20–21,41]. In addition, the benzimidazolidine derivative, BIH (1,3-dimethyl-2-phenyl-benzo[*d*]imidazolidine) (shown in Figure 1) suited well as a sacrificial electron donor, because of its high reducing power [47]. The photocatalytic experiments were performed under 420 nm light irradiation unless otherwise specified. Gaseous products were determined by a gas chromatograph equipped with two barrier discharge ionization detectors (GC–BID). Typically, the concentrations used for the first screening were: 1 mM for PS, 0.1 mM for catalyst **1**, and 20 mM for BIH, and the bases used were either triethanolamine (TEOA) or triethylamine (TEA), see Table 2. Although in the literature some cobalt-based catalysts performed well in acetonitrile [48], our system functioned poorly in CH_3_CN/TEOA 5:1 (v/v), producing only 0.2 μmol of CO and 2.5 μmol of H_2_. Thus, we changed the mixture of solvents, using *N,N*-dimethylacetamide (DMA) as the major component. Although this solvent has very similar properties as the mostly used *N,N*-dimethylformamide (DMF), it is highly stable and does not produce formate upon hydrolysis [49]. In the solvent system DMA/TEOA 3:1 (v/v), we could observe that carbon monoxide was formed, however, the system produced preferentially molecular hydrogen (Table 2, entry 2).

**Table 2:** Selectivity study of photocatalytic CO_2_ reduction with complex **1** as the catalyst.^a^

Entry	Solvent	[BIH], mM	CO, μmol	H_2_, μmol	TON_CO_	Sel._CO_

1	CH_3_CN/TEOA 5:1	20	0.2	2.5	0.5	6.5%
2	DMA/TEOA 3:1	20	0.9	8.5	2.2	10%
3	DMA/TEOA 5:1	20	2.8	11.1	7.0	20%
4	DMA/TEOA 7:1	20	1.1	1.6	2.7	40%
5	DMA/TEA 5:1	20	4.2	0.9	10.4	83%
6	DMA/TEA 7:1	20	3.8	0.1	9.6	97%
7	DMA/TEA 7:1	60	3.9	0.5	9.7	88%
8	DMA/TEA 7:1	80	3.8	0.1	9.6	97%

^a^In a 20 mL flask, 4 mL of solution with the following concentrations, PS (1 mM), complex **1** (0.1 mM) was irradiated at 420 nm for 4 h. Every entry is an average value of at least two tests.

The role of TEOA was studied thoroughly. In many cases, it is a suitable electron donor [47], however, for PS such as Cu(dmp)DPEPhos a higher reducing power is needed. Besides that, TEOA works not only as a Brønsted base (helping in the deprotonation of the radical cation BIH^•+^ formed after the reductive quenching of the PS), but also can actively assist the catalysis, by capturing CO_2_ [50–52]. On the other hand, having three hydroxy groups, TEOA is also considered a proton donor and the formation of metal hydrides is possible. In some cases, this metal hydride favors the production of formate [51]. However, it may induce the concomitant formation of H_2_. This might have been the case of the photo-driven catalysis by complex **1** in DMA/TEOA (Table 2, entries 2–4), where upon decreasing the concentration of TEOA down to 12.5%, the selectivity towards CO increased up to 40%. Nevertheless, H_2_ was still the major product. Thus, we decided to use triethylamine instead of TEOA, since a base is necessary for the reduction of CO_2_, as also demonstrated by control experiments (Supporting Information File 1, Table S4), where in the absence of a base, only little amounts of CO were formed. With TEA, the photocatalytic system generates carbon monoxide with a turnover number (TON) of circa 10. A better selectivity (up to 97%) was achieved when 12.5% of TEA were used (Table 2, entry 6) while increasing the concentration of the electron donor BIH did not result in any increase in the performance (Table 2, entries 7 and 8).

Furthermore, different concentrations of catalyst **1** were evaluated (Table 3). By lowering the concentration of the catalyst, we increased the number of PS molecules per catalyst, resulting in a more efficient electron transfer and consequently an enhancement of the TON. In some cases, the production of H_2_ was too low to be detected by our instrumentation, so we can affirm that the selectivity is higher than 97%, measured in previous cases. A maximum efficiency could be reached with 5 μM of **1**, which produced CO with a TON ≈ 61 after 4 h (Table 3, entry 5). Longer irradiation times (15 h) were evaluated for the concentration of 10 μM and 5 μM of complex **1**, showing that the catalysis continued beyond 4 hours and reached a TON higher than 80 and 50, for [**1**] of 5 and 10 μM, respectively (Table 3, entries 6 and 7).

**Table 3:** Photocatalytic CO_2_ reduction tests with different concentrations of **1**^a^.

Entry	[**1**], mM	Cat., μmol	CO, μmol	TON_CO_

1	0.1	0.5	4.2	8.4
2	0.05	0.25	4.0	16.0
3	0.025	0.125	2.8	22.5
4	0.01	0.05	1.4	27.8
5	0.005	0.025	1.5	60.9
6^b^	0.005	0.025	2.15	86.0
7^b^	0.01	0.05	2.75	53.0

^a^In a 20 mL flask, 5 mL of a solution with the following concentrations, PS (0.5 mM), BIH (20 mM) and complex **1** at the given concentrations was irradiated for 4 h. Every entry is an average value of at least two tests. ^b^Irradiation time was 15 h.

**Figure 5:**
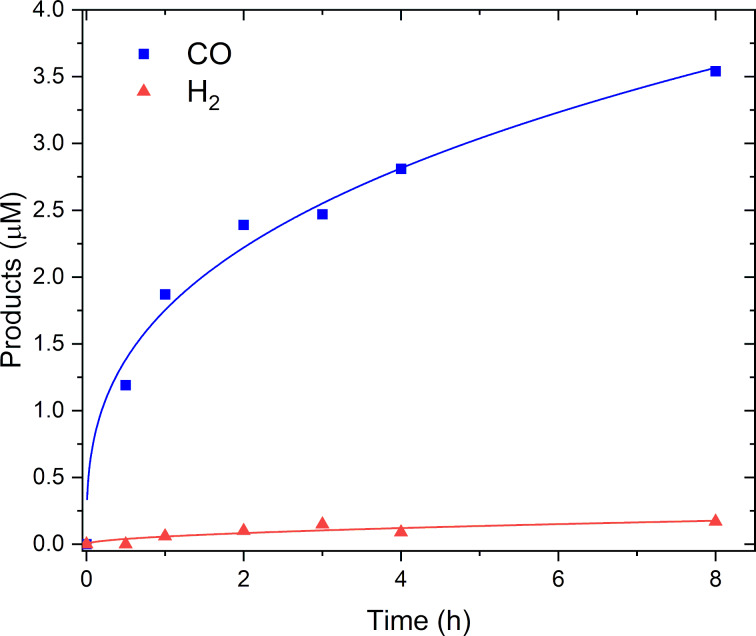
Time evolution of CO (blue squares) and H_2_ (red triangles) with the power functional fitting (blue and red curve, respectively). Data were collected for photocatalytic tests in 5 mL DMA/TEA 7:1, [PS] = 0.5 mM; [**1**] = 0.025 mM; [BIH] = 10 mM.

The reaction kinetics was evaluated in photocatalytic systems containing 0.025 mM of **1**, since the amount of produced CO should be sufficient to be detected by GC-BID, even after a very short time from starting the reaction. As shown in Figure 5, in the first four hours, the formation of CO presents a power functional profile, without any induction period. Moreover, the turnover frequency (TOF) is maximum at 0.5 h, with a value of 19 h^−1^. The catalysis continues; however, the TOF is decreased considerably.

Redox potentials were measured in the same solvent mixture as the photocatalytic tests (DMA/TEA 7:1), to study the thermodynamics of the reaction. The plots are shown in Supporting Information File 1 (Figures S5–S7) and the values are reported versus Me_10_Fc, as the internal standard (Table S3). The first reduction of the cobalt-based catalyst is −1.56 V, thus the electron donation from PS^−^ (−1.67 V) is plausible, albeit the difference is not high. Estimation of the redox potentials of the excited state of PS (E_ox_* and E_red_*) was done assuming that the energy difference (*E*_00_) between the energies of the excited and ground states, both at their zero levels, is the same as the emission maximum. Being the emission of [Cu(dmp)DPEPhos](BF_4_) in DMA/TEA 7:1 567 nm, the value of *E*_00_ is 2.19 eV. It follows that *E*_ox_* is −1.22 V and *E*_red_* is 0.52 V (see Table S3 in Supporting Information File 1). Thermodynamically, an oxidative quenching of PS* by the catalyst **1** is not feasible *(*Δ*G* > 0.3 V), while a reductive quenching by BIH could be possible since the oxidation potential of BIH is 0.27 V (Δ*G* < −0.25 V).

We performed Stern–Volmer analyses to verify our hypothesis. As expected, the lifetime of the PS* (τ_0_ = 14 ns in aerated DMA/TEA 7:1), is reduced upon the addition of the sacrificial electron donor (Figure S13 in Supporting Information File 1). The quenching constant, calculated from the linear fit according to the Stern–Volmer equation, is 3.7 × 10^9^ s^−1^ M^−1^. Thus, the reductive quenching of the photoexcited PS* by BIH is thermodynamically and kinetically feasible. The changes in the UV–vis absorption of a typical photocatalytic solution under irradiation were monitored over a period of four hours (Figure S12 in Supporting Information File 1) and the spectra show the development of a new broad band at 590 nm, reaching its maximum intensity after 2.5 h. This could be due to the accumulation of the reduced PS^−^ species.

**Scheme 1:**
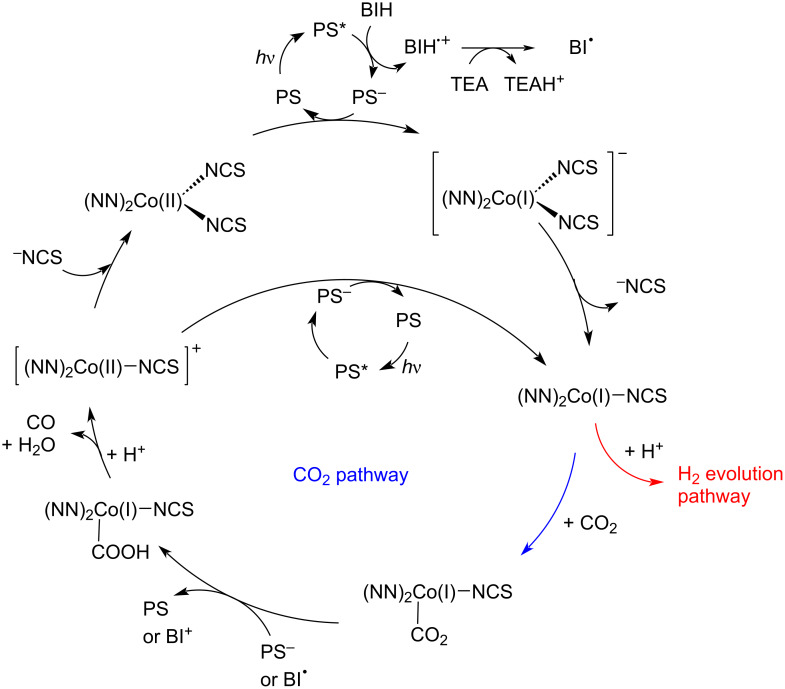
Proposed mechanism for the photoinduced reduction of carbon dioxide with the system presented in this work.

We propose the following mechanism (Scheme 1). The PS absorbs a photon (420 nm) and in its excited state is quenched by BIH, which is deprotonated by the base (TEA) and forms a radical (BI**^·^**). Since this radical is highly reducing, it can happen that this species can also serve as a reductant [47]. The reduced species PS^−^ can be oxidized back to PS by a molecule of **1**, which could detach a ^−^NCS anion and offer a vacant site to coordinate a proton (then following an H_2_ evolution path) or a molecule of CO_2_ [46]. The adduct with CO_2_ is further reduced (by PS^−^ or BI**^·^**) and after the addition of two protons, CO and H_2_O are produced.

The cycle could be closed by the coordination of thiocyanate to the cationic Co(II) species, which is left after the generation of the products, or maybe the reduction of the above-mentioned species occurs with another PS cycle. This proposed mechanism is only tentative and should be confirmed by further analyses and theoretical calculations.

In any case, the addition of proton sources should be beneficial for the CO_2_ pathway, and in the system, we studied so far, the only plausible proton source is the bezimidazolidine derivative. Thus, aiming at enhancing the catalytic activity, we performed some additional photocatalytic tests, upon the addition of different concentrations of 1,1,1,3,3,3-hexafluoropropan-2-ol (HFIP). This alcohol has interesting physical and chemical properties, and, being well miscible with many organic solvents and with water, it has been used in a large variety of (electro)chemical reactions [53]. The hydroxy group of this alcohol has a p*K*_a_ of 9.3 [54–55], so we can expect that it is a suitable proton donor for this kind of reaction. We performed the photocatalytic CO_2_ reduction by dissolving in 5 mL 1%, 2%, and 5% of HFIP (see Table 4). The concentrations of the main components were: [**1**] = 5 μM, [PS] = 0.5 mM, and [BIH] = 10 mM. After four hours of irradiation at 420 nm, the production of CO increased remarkably, reaching a TON higher than 230 when 5% HFIP were used (Table 4, entry 3). Unfortunately, also the generation of H_2_ increased with the concentration of HFIP, lowering the selectivity to 55%. Nevertheless, these results are promising, and further optimization studies are necessary to achieve high efficiencies and selectivity at the same time.

**Table 4:** Photocatalytic CO_2_ reduction tests with different concentrations of HFIP.^a^

Entry	HFIP, %	CO, μmol	H_2_, μmol	TON_CO_	TON_H2_	Sel._CO_

1	1	4.6	3.1	184	126	59%
2	2	4.9	2.1	198	83	70%
3	5	6.0	4.7	231	189	55%

^a^In a 20 mL flask, 5 mL of a solution with the following concentrations, PS (0.5 mM), BIH (20 mM), complex **1** (0.005 mM) and the indicated amounts of HFIP was irradiated for 4 h.

## Conclusion

We presented a novel Co(II)-based catalyst and its employment in photo-driven CO_2_ reduction. The cobalt core was hexacoordinated by two chelating quinolyl-triazole ligands and two ^−^NCS groups. The electrochemical properties suggested that this complex could reduce carbon dioxide. The photocatalytic system chosen for this target was fully earth-abundant, as the complex [Cu(dmp)DPEPhos](BF_4_) was used as the photosensitizer. Preliminary tests in the solvent mixture of DMA/TEOA showed that the novel catalyst reduces CO_2_ to CO. However, the evolution of molecular hydrogen was prevailing. Thus, we modified the conditions to switch the selectivity towards the two electron-reduction product of CO_2_, carbon monoxide. We successfully achieved a selectivity of 97% with the use of TEA (12.5%) instead of TEOA. The following optimization studies allowed us to tune the efficiency for CO production with a maximum TON of 86, after 15 h of irradiation. Finally, further tests were performed with the addition of an additional proton source (HFIP). Although the selectivity was lowered, the CO evolution was enhanced remarkably, reaching a TON up to 230, after 4 h of irradiation.

## Experimental

### Synthesis of catalyst **1**, (BzQuTr)_2_Co(NCS)_2_

In a two-necked round-bottomed flask, under argon, the chelating ligand BzQuTr (100 mg, 0.35 mmol, 2.0 equiv) dissolved in 10 mL of dry MeOH was added dropwise to Co(NCS)_2_(py)_4_ (86 mg, 0.175 mmol, 1.00 equiv), dissolved in 5 mL of MeOH. The mixture was stirred for two hours at room temperature. The solvent was removed under reduced pressure and the crude product was washed with cold MeOH and Et_2_O, obtaining a lilac precipitate (82 mg, 0.11 mmol, 60%). Paramagnetic properties were estimated by the Evans method [56] in acetonitrile and resulted in three unpaired electrons. ATR–IR (cm^−1^) ν: 3109, 3027, 2065, 1606, 1574, 1507, 1496, 1469, 1453, 1438, 1431, 1375, 1358, 1350, 1332, 1313, 1251, 1210, 1162, 1145, 1130, 1101, 1061, 1027, 1011, 952, 832, 817, 803, 783, 764, 732, 717, 694, 679, 654, 636, 599, 572, 531, 516, 482, 459, 399, 384; ESIMS *m*/*z* (%): 689.14 [M − NCS] (100%); 690.15 [M + H − NCS] (43%); Anal. calcd for C_38_H_28_CoN_10_S_2_·CH_3_OH: C, 60.07; N, 17.96; H, 4.11; S, 8.22; found: C, 60.05; N, 17.84; H, 3.66; S, 8.18.

### Photocatalytic CO_2_ reduction

Typically, the tests were performed in glass vials (20 mL) equipped with a screw-cap septum. The solutions were prepared under air and CO_2_ (or argon) was bubbled inside for at least 10 minutes. TEA or TEOA was distilled twice before use. Experiments were performed in a photoreactor from Luzchem (model: LZC-ICH2) equipped with two lamps at 420 nm (fluorescent lamps of 8 W each) and four mini-stirrers. On each stirrer, two samples were irradiated at the same time, for a total of eight simultaneous reactions. A drawing of the photoreactor is shown in the Supporting Information File 1 (Figure S9) and the emission spectrum of the lamp is reported in Figure S10. Typically, the solutions contained the photosensitizer (1 mM or 0.5 mM), catalyst **1** (different concentrations were studied), and BIH (usually 10 mM or 20 mM), unless otherwise noted. The temperature of the reactor was controlled with an in-built ventilator, *T* = (25 ± 5) °C. The moles of products (CO and H_2_) were measured by quantitative analyses of the headspace of the reactions with a gas chromatograph from Shimadzu (GC-2030) equipped with two barrier discharge ionization detectors (BID). Every test was repeated at least twice. The photon flux was evaluated with actinometry, according to a previously published procedure [42], and it was 0.025 µE s^−1^. Therefore, an apparent photoluminescent quantum yield could be estimated to be up to 2.4%, after 4 h, according to Equation 5:


[5] $$\Phi =\frac{\text{CO moles}}{\text{incident photons}\times (1-10^{\text{A}})}\times 100$$

Where A is the initial absorption value of the photocatalytic system at the irradiation wavelength.

TON and TOF were calculated according to Equation 6, Equation 7, and Equation 8:


[6] $${\text{TON}}_{\text{CO}}=\frac{n_{\text{CO}}}{n_{\text{catalyst}}}$$

[7] $${\text{TON}}_{\text{H2}}=\frac{n_{\text{H2}}}{n_{\text{catalyst}}}$$

[8] $$\text{TOF}=\frac{\text{TON}}{t_{\text{reaction}}}$$

Where *n* is the number of moles of the products and of the catalyst; *t* is the time of the reaction.

## Supporting Information

Additional information regarding the instrumentation, structural analyses, and X-ray structures is provided. Crystal structures were deposited in the Cambridge Crystallographic Data Centre (CCDC) with the numbers 2285968 (**1a**) and 2285968 (**1b**).

File 1 General information, further synthetic and experimental procedures, and additional results.
