# Supplementary material for: Selectivity control towards CO versus H2 for photo-driven CO2 reduction with a novel Co(II) catalyst
Source: Beilstein J Org Chem. 2023 Nov 17;19:1766–75. doi: 10.3762/bjoc.19.129 (PMC10667713; doi:10.3762/bjoc.19.129)
Supplement: File 1 — General information, further synthetic and experimental procedures, and additional results. [file Beilstein_J_Org_Chem-19-1766-s001.pdf]

BEILSTEIN JOURNAL OF ORGANIC CHEMISTRY

Supporting Information

for

Selectivity control towards CO versus H_2 for photo-driven CO_2 reduction with a novel Co(II) catalyst

Lisa-Lou Gracia, Philip Henkel, Olaf Fuhr and Claudia Bizzarri

Beilstein J. Org. Chem. **2023,** *19,* 1766–1775. doi:10.3762/bjoc.19.129

General information, further synthetic and experimental procedures, and additional results

License and Terms: This is a supporting information file under the terms of the Creative Commons Attribution License (https://creativecommons.org/licenses/by/4.0). Please note that the reuse, redistribution and reproduction in particular requires that the author(s) and source are credited and that individual graphics may be subject to special legal provisions.
The license is subject to the *Beilstein Journal of Organic Chemistry* terms and conditions: (https://www.beilstein-journals.org/bjoc/terms)

General information

The starting materials, solvents, and reagents were purchased from commercial suppliers and used without further purification unless stated. Anhydrous *N,N*-dimethylacetamide (<0.005% water) was purchased over molecular sieves, while triethylamine (TEA) and triethanolamine (TEOA) were purified by distillation. For the photocatalytic tests, 20 mL-glass vials were purchased from TH.Geyer, with a septum. NMR spectra were recorded on a Bruker Advance 400 NMR instrument at 400 MHz for ^{1}H NMR and 126 MHz for ^{13}C NMR. The NMR spectra were recorded at 22 °C in deuterated solvents. Electron spray ionization (ESI) experiments were recorded on a Thermo Fisher Scientific Q-Exactive (Orbitrap) mass spectrometer, at the Institute of Organic Chemistry (Karlsruhe Institute of Technology).

Syntheses

Photosensitizer [Cu(dmp)DPEPhos](BF_4)

The synthesis of the heteroleptic Cu(I) complex used as photosensitizer was performed according to the general procedure, already published [1]. In a Schlenk tube, under an inert atmosphere (Ar) a solution of DPEPhos and $Cu(CH_3CN)_4BF_4$ in dry CH_2Cl_2 was stirred for 0.5 h at room temperature. One equivalent of 2,9-dimethyl-1,10-phenanthroline was added and the reaction mixture was stirred at room temperature for an additional 5 h. The solvent was removed under vacuum and the residue was purified by column chromatography on silica gel (eluent: CH_2Cl_2; R_f: 0.39) to give the desired compound as a yellow powder with 67% yield. ^{1}H NMR (400 MHz, $CDCl_3$) δ 8.35 (d, *J* = 8.3 Hz, 2H), 7.84 (s, 2H), 7.58 (d, *J* = 8.3 Hz, 2H), 7.34 (td, *J* = 8.2, 7.8, 1.9 Hz, 3H), 7.23 – 7.14 (m, 7H), 7.02 (t, *J* = 7.4 Hz, 8H), 6.98 – 6.89 (m, 12H), 2.43 (s, 6H).

BIH

The synthesis of the sacrificial electron donor was pursued using a strategy from the literature [2]. The product was recrystallized at least twice before employment. As starting material, 2-phenyl-1*H*-benzo[*d*]imidazole was commercially supplied by Merck. ^{1}H NMR: (400 MHz, $CDCl_3$): δ (ppm) 7.63–7.56 (m, 2H), 7.42 (dd, ^1J = 5.1 Hz, ^2J = 1.8 Hz, 3H), 6.73 (dd, ^1J = 5.5 Hz, ^2J = 3.2 Hz, 2H), 6.45 (dd, ^1J = 5.4 Hz, ^2J = 3.1 Hz, 2H), 4.89 (s, 1H), 2.50 (s, 6H).

Ligand

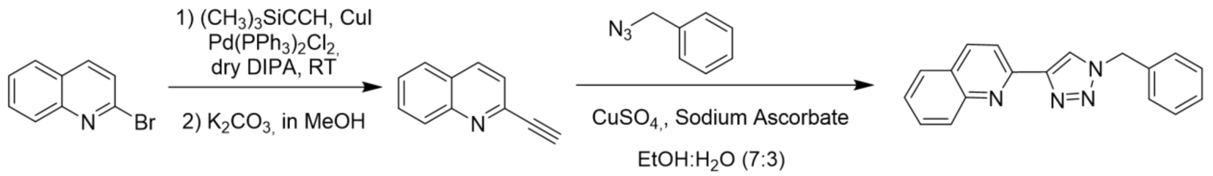

The synthesis of the ligand for the catalyst was achieved in two steps following the experimental procedure published previously [3]. Caution must be taken when handling organic azides as they might be explosive.

2-Ethynylquinoline was obtained with an 82% yield. ^{1}H NMR: (400 MHz, $CDCl_3$): δ (ppm) 8.12 (t, *J* = 8.4 Hz, 2H), 7.80 (d, *J* = 8.0 Hz, 1H,), 7.73 (ddd, ^{1}J = 8.6 Hz, ^{2}J = 6.9 Hz, ^{3}J = 1.5 Hz, 1H), 7.55 (dd, ^{1}J = 9.0 Hz, ^{2}J = 6.0 Hz, 2H), 3.25 (s, 1H). ^{13}C NMR: (126 MHz, $CDCl_3$): δ (ppm) 148.11, 142.42, 136.29, 130.20, 129.48, 127.48, 127.44, 124.16, 83.38, 77.66. MS (ESI): *m/z* (%) = 153.10 [M] (100); HRMS ($C_{11}H_7N$): calc.: 153.0578, found: 153.1290.

1-Benzyl-4-(quinolin-2-yl)-1*H*-1,2,3-triazole (BzQuTr) was obtained as a beige solid in 93% yield. ^{1}H NMR: (400 MHz, $CDCl_3$): δ (ppm) 8.34 (d, *J* = 8.0 Hz, 1H), 8.26 (s, 1H), 8.24 (d, *J* = 8.0 Hz, 1H), 8.00 (d, *J* = 8.0 Hz, 1H), 7.81 (d, *J* = 8.0 Hz, 1H), 7.69 (ddd, ^{1}J = 8.5 Hz, ^{2}J = 6.9 Hz, ^{3}J = 1.5 Hz, 1H), 7.51 (ddd, ^{1}J = 8.1 Hz, ^{2}J = 6.9 Hz, ^{3}J = 1.2 Hz, 1H), 7.40–7.35 (m, 5H,), 5.63 (s, 2H). ^{13}C NMR: (126 MHz, $CDCl_3$): δ (ppm) 150.49, 149.11, 148.05, 136.87, 134.42, 129.75, 129.21, 129.00, 128.91, 128.34, 127.81, 127.78, 126.36, 122.69, 118.71, 54.48; MS (ESI): *m/z* (%) = 287.13 [M+H] (43); HRMS ($C_{18}H_{14}N_4$): calc.: 286.1218, found: 287.1320

Cobalt precursor $Co(NCS)_2(py)_4$

Cobalt perchlorate, $Co(ClO_4)_2{\cdot}6H_2O$ (1.2 g; 3.28 mmol, 1.00 equiv) was dissolved in 20 mL water. In a separate flask, NH_4SCN (500 mg; 6.56 mmol, 2.00 equiv) and 1.35 mL of pyridine (py, 1.3 g,16.4 mmol, 5.00 equiv) were dissolved in 15 mL H_2O and the solution was added dropwise into the cobalt perchlorate solution. The reaction mixture was stirred for thirty minutes at room temperature during which a pink precipitate was formed. It was filtered off and washed with EtOH/pyridine 9:1 and dried under a high vacuum to give the desired precursor (1.3 g, 2.62 mmol, 76%). MS (ESI): *m/z* (%) = 354.03 [M-py-NCS] (100); HRMS ($C_{22}H_{20}CoN_6S_2$): calc.: 491.0523, found: 354.0342.

Cobalt catalyst, 1

In a two-necked round-bottomed flask, under argon, the chelating ligand BzQuTr (100 mg, 0.35 mmol, 2.0 equiv) dissolved in 10 mL of dry MeOH was added dropwise to a solution of $Co(NCS)_2(py)_4$ (86 mg, 0.175 mmol, 1.00 equiv) in 5 mL of MeOH. The mixture was stirred for two hours at room temperature. The solvent was removed under reduced pressure and the crude product was washed with cold MeOH and Et_2O, obtaining a lilac precipitate (82 mg, 0.11 mmol, 60%). MS(ESI): *m/z* (%)= 689.14 [M − NCS] (100%); 690.15 [M +H − NCS] (43%). IR (ATR): ν (cm^{-1}) = 3109, 3027, 2065, 1606, 1574, 1507, 1496, 1469, 1453, 1438, 1431, 1375, 1358, 1350, 1332, 1313, 1251, 1210, 1162, 1145, 1130, 1101, 1061, 1027, 1011, 952, 832, 817, 803, 783, 764, 732, 717, 694, 679, 654, 636, 599, 572, 531, 516, 482, 459, 399, 384. Anal. calcd for $C_{38}H_{28}CoN_{10}S_2•CH_3OH$: N: 17.96, C: 60.07, H: 4.11, S: 8.22, Found: N: 17.84, C: 60.05, H: 3.66, S: 8.18.

X-ray analysis, experimental details

Single crystal X-ray diffraction data were collected on a STADI VARI diffractometer with monochromated Ga Kα (1.34143 Å) radiation at low temperature (150 K for **1a** and 180 K for **1b**). Using Olex2 [4], the structures were solved with the ShelXT [5] structure solution program using Intrinsic Phasing and refined with the ShelXL [6] refinement package using least squares minimization. Refinement was performed with anisotropic temperature factors for all non-hydrogen atoms; hydrogen atoms were calculated on idealized positions. Crystallographic and structure refinement data of the two polymorphs of **1** (**1a** and **1b**) are summarized in Table S1.

Crystallographic data for compounds **1a** and **1b** reported in this paper have been deposited with the Cambridge Crystallographic Data Centre as supplementary information no. CCDC-2285968 and 2285969. Copies of the data can be obtained free of charge from https://www.ccdc.cam.ac.uk/structures.

Table S1: Crystallographic and structure refinement data of polymorphs of **1**.

Compound	1a	1b
Empirical formula	$C_{38}H_{28}CoN_{10}S_2·(2CH_3CN)$	$C_{38}H_{28}CoN_{10}S_2·(CH_3CN)$
Formula weight	829.86	788.81
Temperature/K	150	180
Crystal system	triclinic	orthorhombic
Space group	$P\bar{1}$	$Pna2_1$
a/Å	9.2762(4)	27.041(3)

b/Å	14.7141(6)	15.0454(10)
c/Å	15.4774(6)	9.2555(6)
α/°	74.890(3)	90
β/°	85.542(3)	90
γ/°	86.397(3)	90
Volume/$Å^3$	2031.33(15)	3765.5(5)
Z	2	4
ρ_{calc}g/cm^3	1.357	1.391
μ/mm^{-1}	3.150	3.375
F(000)	858.0	1628.0
Crystal size/mm^3	0.14 × 0.12 × 0.03	0.08 × 0.03 × 0.02
Radiation	GaKα (λ = 1.34143)	GaKα (λ = 1.34143)
2Θ range for data collection/°	5.156–125.104	5.848–109.992
Reflections collected	27488	18621
Independent reflections	9417 [R_{int} = 0.0256]	5020 [R_{int} = 0.0609]
Ind. refl. with I≥2σ (I)	8271	3802
Data/restraints/parameters	9417/0/516	5020/1/488
Goodness-of-fit on F^2	1.054	0.971
Final R indexes [I≥2σ (I)]	R_1 = 0.0368, wR_2 = 0.0947	R_1 = 0.0554, wR_2 = 0.1297
Final R indexes [all data]	R_1 = 0.0433, wR_2 = 0.0987	R_1 = 0.0817, wR_2 = 0.1405
Largest diff. peak/hole / e $Å^{-3}$	0.54/–0.51	1.14/–0.48
Flack parameter		–0.009(9)
CCDC number	2285968	2285969

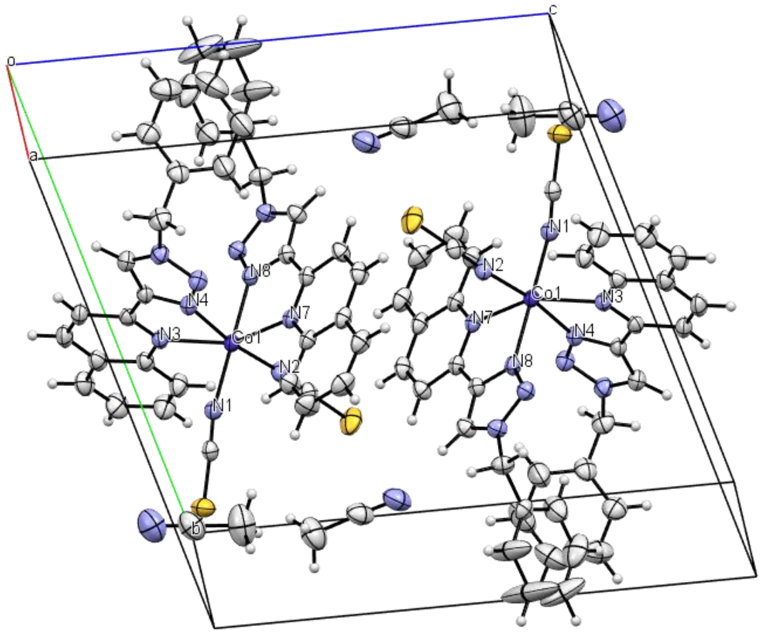

Figure S1: Crystal packing with solvent molecules of polymorph **1a**.

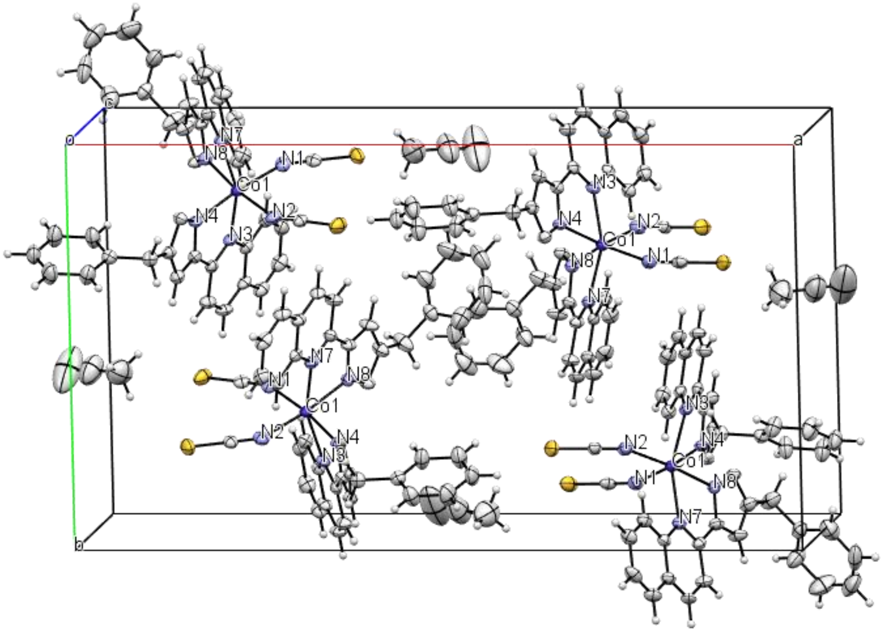

Figure S2: Crystal packing with solvent molecules of polymorph **1b**.

Table S2: Selected bond lengths and angles of polymorphs **1a** and **1b**.

	**1a**	**1b**
Co-N1	2.0611(15) Å	2.085(6) Å
Co-N2	2.0566(14) Å	2.081(6) Å
Co-N3	2.2785(13) Å	2.234(5) Å
Co-N7	2.2512(13) Å	2.252 (5) Å
Co-N4	2.1345(13)	2.147(5) Å
Co-N8	2.1351(14)	2.149(5) Å
N7-Co-N8	76.50(5) °	76.27(19)°
N4-Co-N3	75.52(5)°	75.94(19)°
N2-Co-N1	89.39(6)°	86.0(2)°

Infrared spectroscopy (IR) measurement of complex 1

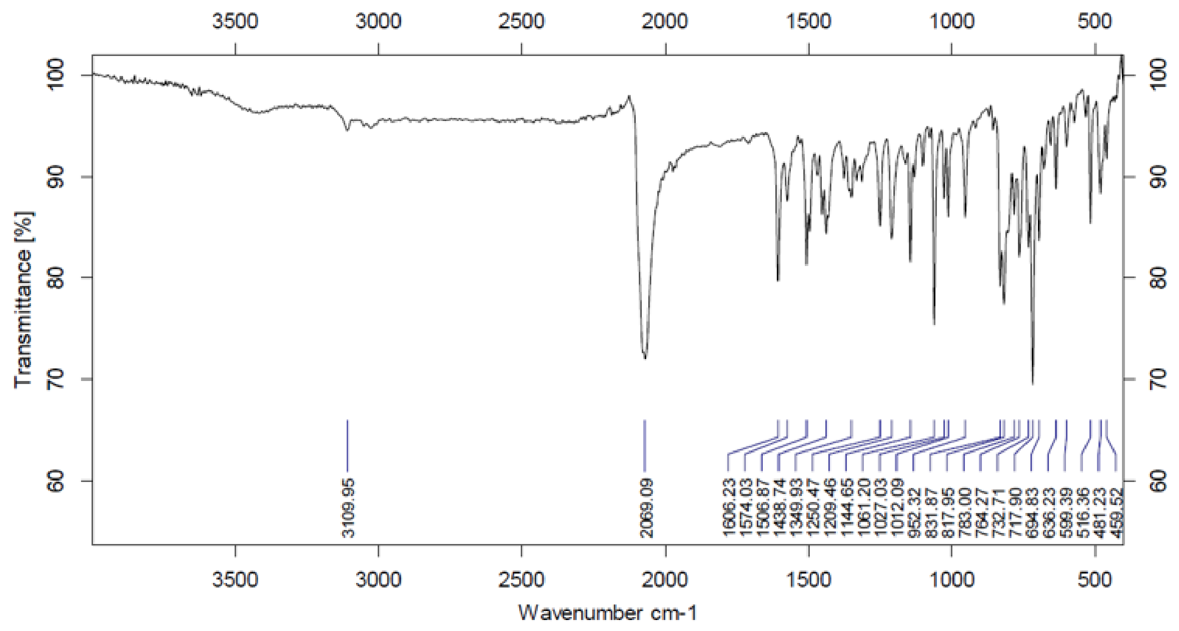

Figure S3: FTIR spectrum of complex **1**, measured by attenuated total reflection (ATR), was recorded with a Bruker Alpha P instrument.

Mass spectrometry (MS) measurement of complex 1

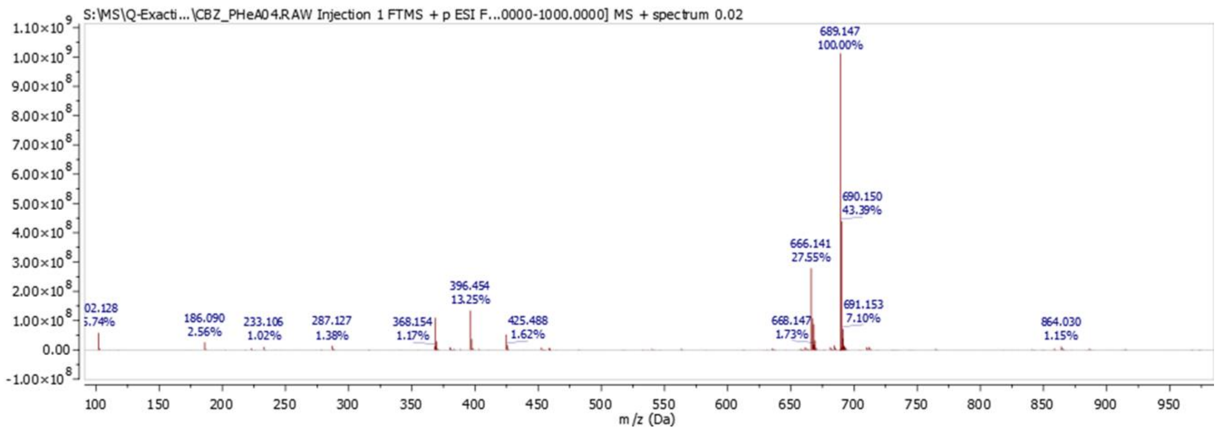

Figure S4. Mass spectrum of **1**, measured with electron spray ionization (ESI), recorded on a Thermo Fischer Scientific Q-Exactive (Orbitrap) mass spectrometer.

Electrochemical measurements

Electrochemical experiments were recorded on a Gamry Interface 1010B potentiostat. The electrochemical cell was equipped with three electrodes: a glassy-carbon disk as a working electrode, a Pt wire as an auxiliary electrode, and a Ag wire as a quasi-reference. Internal standards were necessary to calibrate the potential and ferrocene (Fc) or decamethylferrocene (Me10Fc) were used, according to the electrochemical window. The potentials of Fc and $Me_{10}Fc$ differ by about 450 mV in DMA with our

system. For comparison with the values, please refer to literature sources [7-9]. The experiments were performed using tetrabutylammonium hexafluorophosphate as a supporting electrolyte (0.1 M). Scan rates were typically 100 mV/s.

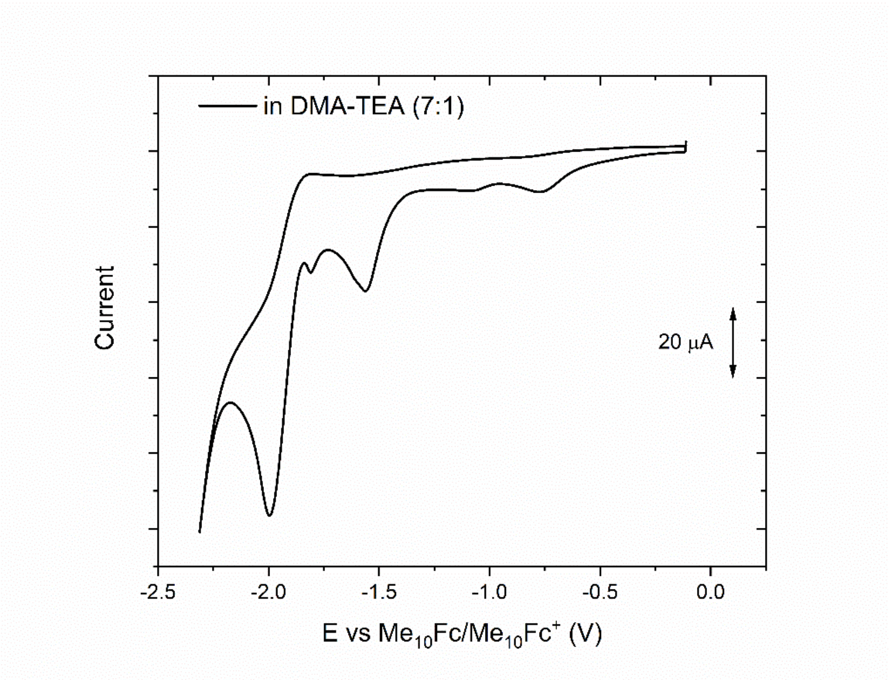

Figure S5: Cyclic voltammogram of **1** in DMA/TEA 7:1 (v/v), 0.1 M $TBAPF_6$, recorded at a scan rate of 100 mV/s. Potentials are reported versus the internal reference $Me_{10}Fc$.

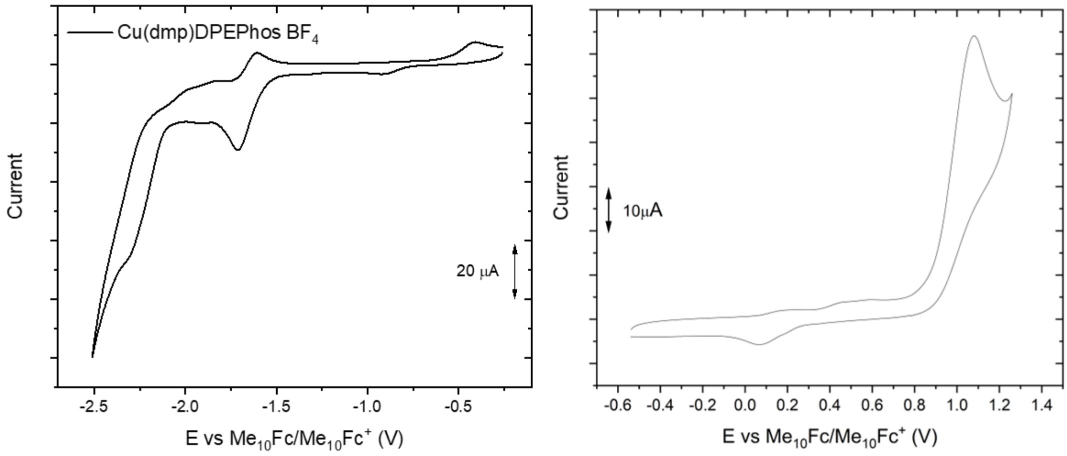

Figure S6: Cyclic voltammograms of $Cu(dmp)DPEPhosBF_4$ in DMA/TEA 7:1 (v/v), 0.1 M $TBAPF_6$, recorded at a scan rate of 100 mV/s. Left: reduction scan and right: oxidation scan. Potentials are reported versus the internal reference $Me_{10}Fc$.

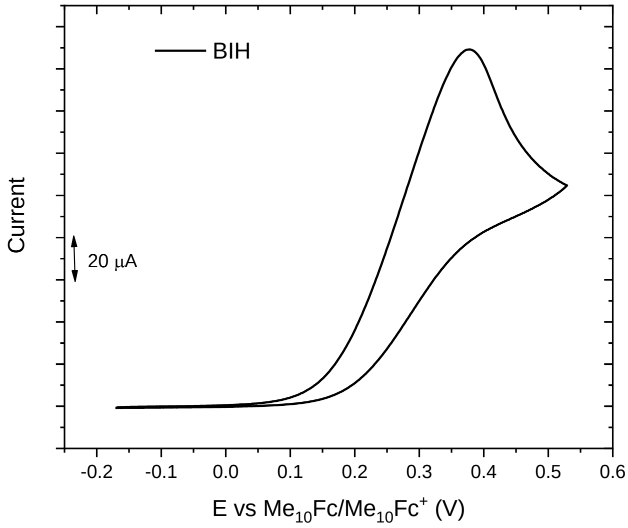

Figure S7: Cyclic voltammogram of BIH in DMA/TEA 7:1 (v/v), 0.1 M $TBAPF_6$, recorded at a scan rate of 100 mV/s. Potentials are reported versus the internal reference $Me_{10}Fc$.

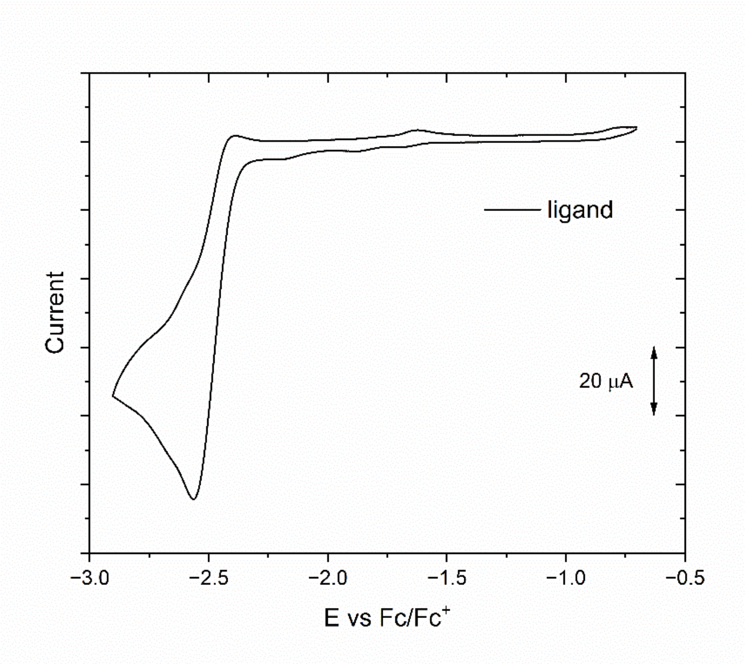

Figure S8: Cyclic voltammogram of ligand in DMA, 0.1 M $TBAPF_6$, recorded at a scan rate of 100 mV/s. Potentials are reported versus the internal reference ferrocene.

Table S3: Electrochemical data in DMA/TEA 7:1 (v/v).

Sample	E_{ox} (V)	E_{red} (V)	E_{ox}* (V)	E_{red}* (V)
Cu[b]	−1.09	−1.67	−1.22	0.52
**1**	–	−1.56;<br>−2.02	–	–
BIH	0.27	–	–	–

[a]Scan rate 100 mV/s; 0.1M TBAPF$_6$. [b]Cu is [Cu(dmp)DPEPhos[(BF$_4$).

Photo-driven CO_2 reduction

Experiments were performed in a photoreactor from Luzchem (model: LZC-ICH2) equipped with four lamps at 420 nm and four mini-stirrers. On each stirrer, two samples were irradiated at the same time, for a total of eight simultaneous reactions. Typically, the solutions contained the photosensitizer (1 mM or 0.5 mM), catalyst **1** (different concentrations studied), and BIH (usually 10 mM or 20 mM), unless otherwise noted. At the irradiation wavelength (420 nm) the optical density was 0.35. The temperature of the reactor was controlled with an in-built ventilator, T = (25 ± 5) °C. The moles of products (CO and H_2) were measured by quantitative analyses of the headspace of the reactions with a gas chromatograph from Shimadzu (GC-2030) equipped with two barrier discharge ionization detectors (BID). Every test was repeated at least twice.

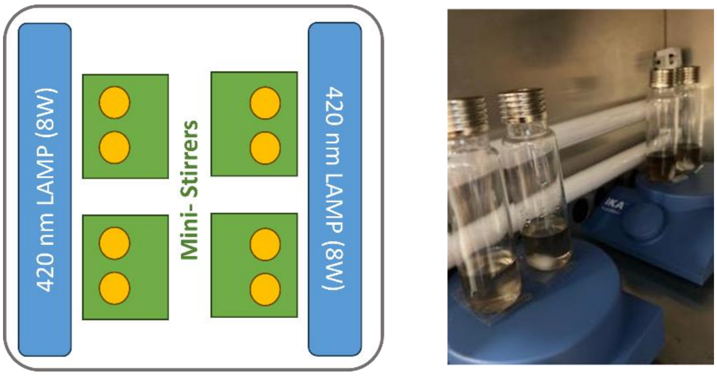

Figure S9: (Left) Explanatory drawing of the set-up, observed from above. In the photoreactor chamber, the samples (yellow circles are put at a distance of 1 cm from the light source on a mini-stirrer (green rectangular shapes). On a mini-stirrer, two photocatalytic systems were put. (Right) A picture of some irradiated samples in the photoreactor, taken after photocatalysis.

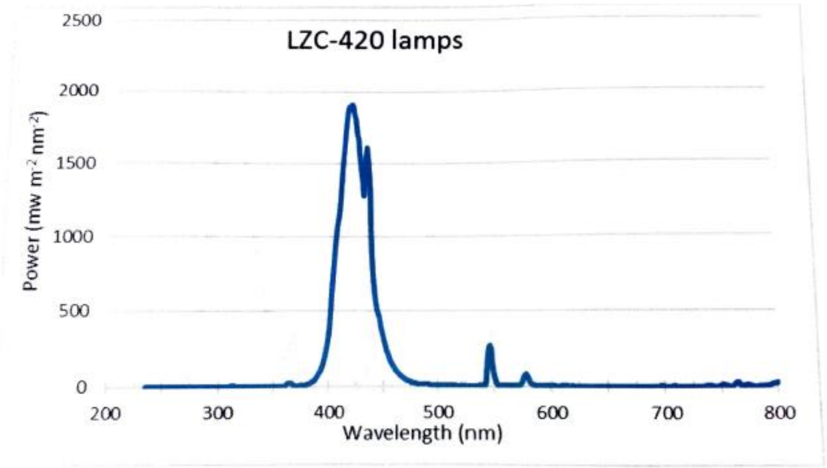

Figure S10: Emission spectrum of the Luzchem lamp at 420 nm.

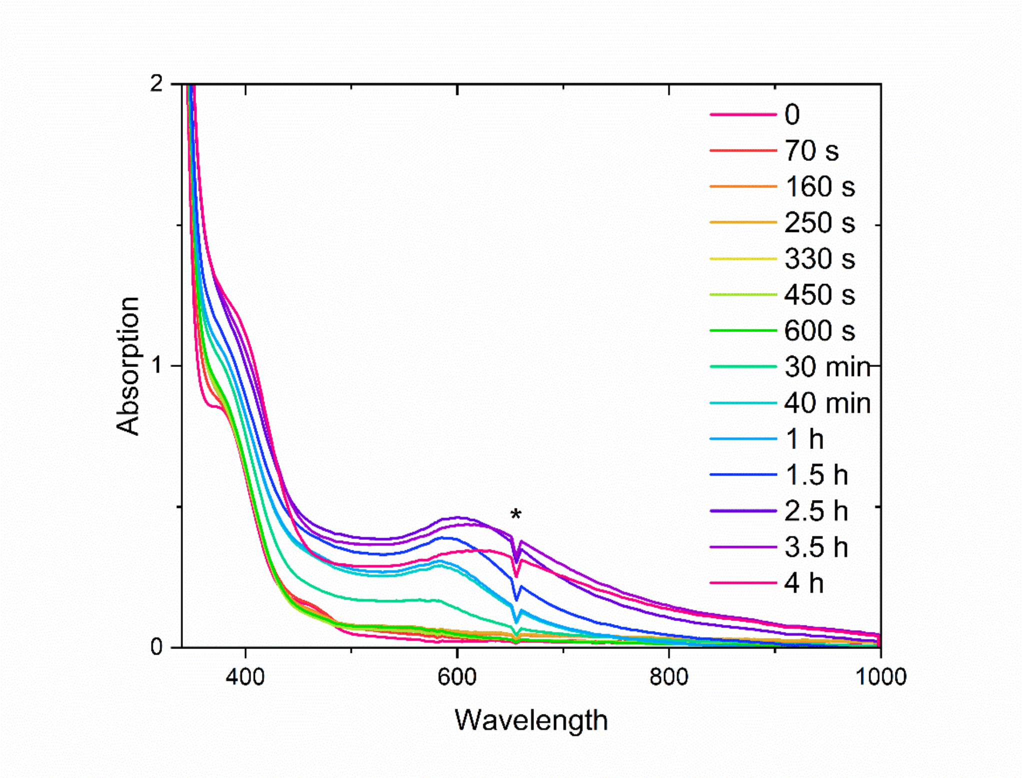

Figure S11: Monitoring of the absorption changes during a typical photocatalysis, DMA/TEA 7:1 (v/v), PS: 1 mM; catalyst 0.01 mM; BIH 10 mM; under irradiation at 420 nm over the period of 4 h. (Asterisk shows the lamp change of the spectrometer).

Table S4: Control photocatalytic CO_2 reduction tests.[a,b]

Entry	Deviation from standard catalysis	BIH (mM)	CO (μmol)	H_2 (μmol)	TON_{CO}	$Sel._{CO}$
1	Without any base	20	0.8	n.d.	1.9	>99%
2	Without any base	40	1.62	n.d.	4.1	>99%
3	With Ar (no CO_2)	20	n.d.	0.8	–	–
4	Without PS	20	n.d.	n.d.	–	–
5	Without **1**	20	n.d.	1.3	–	–

[a]Standard conditions: DMA/base 7:1 (v/v), PS: 1 mM; catalyst 0.1 mM; BIH 20 mM; excitation at 420 nm for 4 h. [b]n.d. = not detected

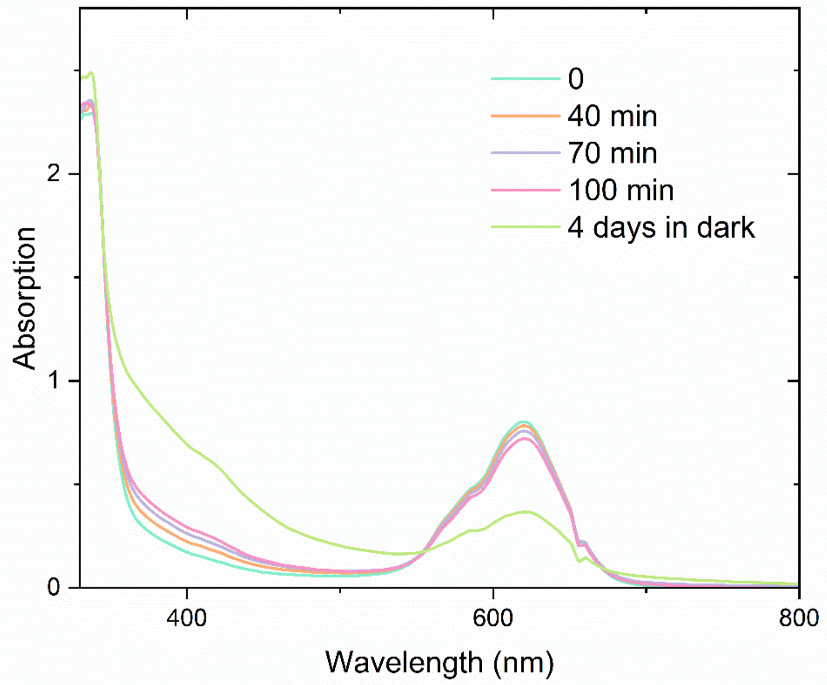

Figure S12: Monitoring of the absorption changes of the catalyst (0.001 M) in a solution DMA/TEA 7:1 (v/v) during the first hundred minutes under irradiation at 420 nm, small decrease of the d–d band is visible, and a slightly increased absorption at around 400 nm can be detected. The solution was left in the dark for 4 days, and the evident spectral changes indicate the instability of complex **1** in the solution after this long timeframe.

Stern–Volmer analysis

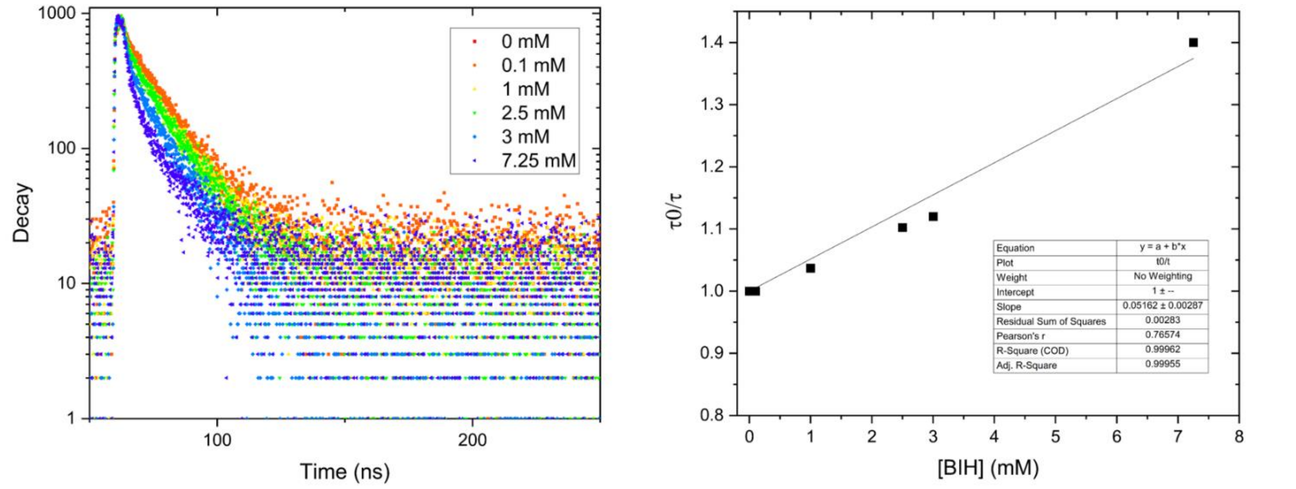

Figure S13: (left) Emission intensity decays (λ_{ex}: 375 nm (NanoLED); λ_{em}: 550 nm) of Cu(dmp)DPEPhosBF_4 (0.25 mM) with different quencher concentrations in air-equilibrated DMA/TEA 7:1 (v/v). (right): Stern–Volmer plot. Fitting curve: y = 0.052x + 1 (R^2: 0.999). $k_q = 3.7 \times 10^9\ s^{-1}\ M^{-1}$.

References.

[1] Cuttell, D. G.; Kuang, S.-M.; Fanwick, P. E.; McMillin, D. R.; Walton, R. A. *J. Am. Chem. Soc.* **2002**, *124*, 6–7.

[2] Stanley, P. M.; Haimerl, J.; Thomas, C.; Urstoeger, A.; Schuster, M.; Shustova, N. B.; Casini, A.; Rieger, B.; Warnan, J.; Fischer, R. A.-*Angew. Chem. Int. Ed.* **2021**, *60*, 17854.

[3] L.-L. Gracia, L. Luci, C. Bruschi, L. Sambri, P. Weis, O. Fuhr, C. Bizzarri, *Chem. Eur. J.* **2020,** *26*, 9929.

[4] O. V. Dolomanov, L.J. Bourhis, R. J. Gildea, J. A. K. Howard, H. Puschmann, *J. Appl. Cryst.* **2009**, *42*, 339–341.

[5] G. M. Sheldrick, *Acta Cryst. A* **2015**, *A71*, 3–8.

[6] G. M. Sheldrick, *Acta Cryst. C* **2015**, *C71*, 3–8.

[7] R. R. Gagne, C. A. Koval, G. C. Lisensky *Inorg. Chem*. **1980**, *9*, 2854-2855.

[8] N. Tsierkezod *J. Solution Chem.* **2007**, *36*, 289–302.

[9] I. Noviandri, K. N. Brown, D. S. Fleming, P. T. Gulyas, P. A. Lay, A. F. Masters, L. Philips *J. Phys. Chem. B.* **1999**, *103*, 6713-6722.
